# Whale-tail–shaped mucocutaneous flap for the reconstruction of electrical burn at the labial commissure: a case report

**DOI:** 10.1097/RC9.0000000000000614

**Published:** 2026-06-26

**Authors:** Daniel Fernando Sastoque Parisier, Adriana Parra Gomez, Sebastian Murcia Espino, Maria Alejandra Gomez Gutierrez, Julián David Rojas Guzman, Juan Camilo Vacca Rubio

**Affiliations:** a Department of Plastic and Reconstructive Surgery, Hospital Simón Bolivar, Bogotá, Colombia; b Founder and leader of the Plastic and Reconstructive Surgery Research Group, Universidad El Bosque, Bogotá, Colombia; c Plastic and Reconstructive Surgery Research Group, Universidad El Bosque, Bogotá, Colombia

**Keywords:** electrical burn, mucocutaneous flap, oral commissure, pediatric burn

## Abstract

**Introduction and importance:**

Electrical injuries in children are uncommon but may cause disproportionate functional and esthetic sequelae, particularly in the perioral region. Even small oral commissure defects can disrupt oral competence, symmetry, and vermilion continuity. We present a pediatric case of a full-thickness labial commissure defect following a low-voltage electrical burn, reconstructed with a local whale-tail–shaped mucocutaneous flap.

**Case presentation:**

A 6-year-old boy sustained a low-voltage electrical burn to the left labial commissure after biting a charger cable. Examination showed a full-thickness commissural defect with incomplete oral closure and commissural distortion. Initial treatment included hydrogel and petrolatum dressings to allow autolytic debridement and better tissue demarcation. Definitive reconstruction was then performed. After escharectomy, a 3 × 1 cm full-thickness defect with orbicularis oris exposure remained. A whale-tail–shaped local mucocutaneous flap was raised from the adjacent cheek to restore muscular continuity, the mucocutaneous junction, the vermilion border, and the commissural angle. At 6 months, the patient showed preserved oral competence, adequate oral aperture, and satisfactory symmetry, with only mild residual asymmetry during mouth opening.

**Clinical discussion:**

Perioral electrical burns may appear limited in surface area but can result in full-thickness injury with major reconstructive implications. In this case, delayed reconstruction after short-term conservative wound care allowed better definition of viable tissue. The local flap provided like-with-like reconstruction in a single stage while avoiding prolonged splinting or staged procedures.

**Conclusion:**

When local tissue is preserved, a whale-tail–shaped mucocutaneous flap may offer effective single-stage reconstruction of acute commissural electrical burns in pediatric patients.

## Introduction

Electrical injuries in children are uncommon but potentially devastating and represent a small proportion of pediatric burn admissions worldwide^[^1,2^]^. Low-voltage injuries usually occur at home in younger children, often from electrical cords or household outlets[2]. Despite limited total body surface area, perioral burns can cause deep tissue injury with major functional and esthetic sequelae^[^1,3^]^.

HIGHLIGHTS

- Low-voltage perioral electrical burns in children can cause full-thickness oral commissure defects with major functional impacts despite the small surface area.
- A short period of conservative wound care (hydrogel/petrolatum) helped demarcate viable tissue and define margins before definitive reconstruction.
- Single-stage “whale-tail” mucocutaneous local flap reconstruction restored orbicularis oris continuity, commissural angle, and vermilion architecture using like-with-like tissue.
- At 6 months, the patient maintained oral competence and adequate oral aperture, with preserved smile dynamics and only mild residual asymmetry during mouth opening.
- This case supports anatomically precise local flap selection as an effective alternative to more complex or staged techniques in pediatric commissure reconstruction.

Perioral electrical injuries can have functional consequences disproportionate to the apparent defect size. The perioral complex is essential for oral competence, speech, mastication, and facial expression^[^4,5^]^. The oral commissure is a specialized region where upper and lower orbicularis oris fibers converge into the modiolus, a conical hub that integrates perioral muscles to preserve sphincter function and symmetry[5]. Therefore, even small commissural defects may cause distortion, microstomia, or oral incompetence.

Multiple techniques have been described for oral commissure defects, ranging from local advancement flaps to cross-lip and tongue flaps, depending on the extent and depth of tissue loss^[^4–6^]^. In the reconstructive elevator paradigm, more complex procedures are often considered for functional restoration, but complexity does not necessarily yield better functional or esthetic outcomes. In pediatric patients, limited cooperation, postoperative compliance, and the potential need for staged procedures further influence surgical planning. We present the case of a pediatric patient with a full-thickness labial commissure defect managed with a local mucocutaneous flap and discuss the rationale supporting this conservative yet anatomically precise reconstructive approach. This case report has been prepared and reported in accordance with the SCARE 2025 guidelines[7].

## Presentation of case and treatment

**Figure 1.**
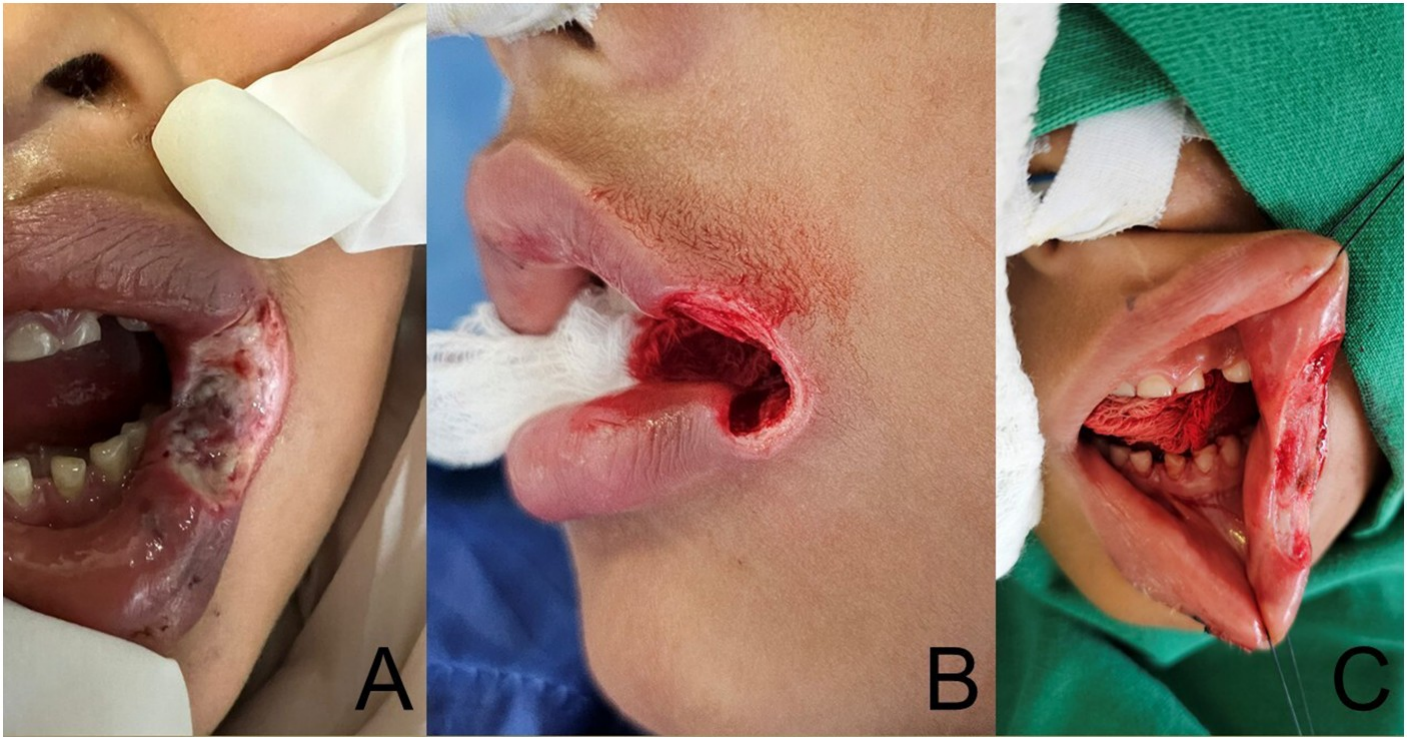
(A) Electrical low-voltage burn affecting the superior and inferior lips, as well as the left oral commissure. (B, C) Resulting defect after escharectomy and border defect debridement.

A 6-year-old male patient was admitted to the burn unit of a tertiary hospital after sustaining a low-voltage electrical injury to the left labial commissure caused by biting a mobile phone charger cable. Evaluation by the plastic and reconstructive surgery department revealed a full-thickness defect involving the labial commissure, resulting in incomplete oral closure and distortion of the commissural subunit (Fig. 1A).

Initial treatment consisted of daily wound care with hydrogel and petrolatum dressings for 7 days, allowing clear identification of nonviable tissue and better definition of the wound margins prior to definitive surgical reconstruction.

After the optimization of the wound bed with autolytic debridement over 7 days, the patient underwent surgical intervention under general anesthesia with nasotracheal intubation. Standard aseptic preparation was performed using chlorhexidine and saline irrigation.

Escharotomy was carried out with a scalpel, including marginal excision of devitalized tissue to regularize the wound edges, facilitating closure with healthy tissue. Hemostasis was achieved using adrenaline-soaked gauze and low-power electrocautery. Following debridement, a residual 3 × 1 cm full-thickness defect with exposure of the orbicularis oris muscle was identified (Fig. 1B and C).

Measurements were obtained from the superior border of the Cupid’s bow to the left commissure to guide reconstruction. An additional 1 cm was incorporated on the affected side to account for possible scar contracture and to achieve symmetry.

**Figure 2.**
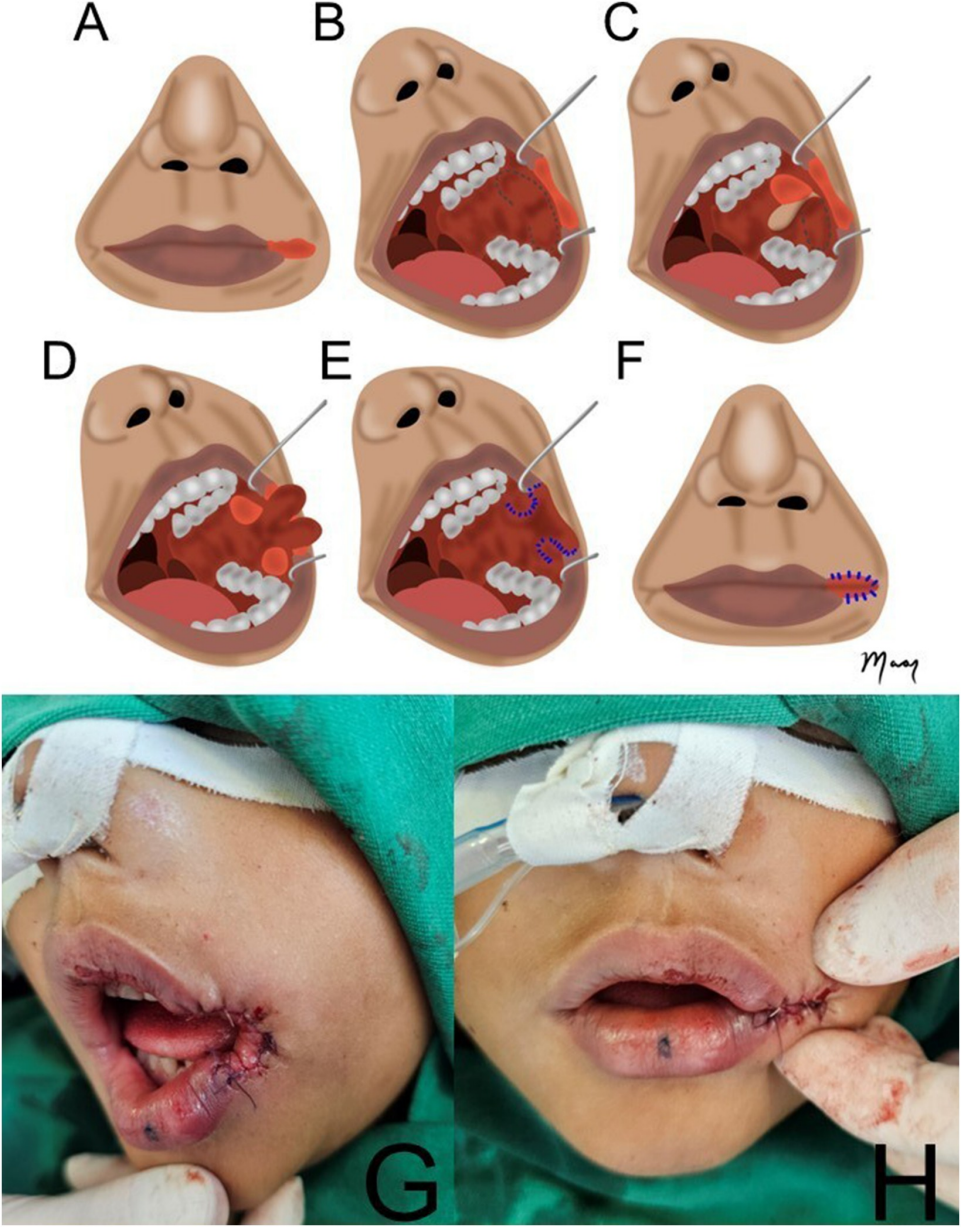
The illustration represents: (A) Left commissure defect. (B) Design and marking of a “whale tail” shape with methylene blue. (C) Lifting the lobes of the flap after incision. (D) Placement of the superior and inferior lobes of the flap. (E) Positioning the flaps with monofilament on the skin areas and multifilament on the mucosal areas. (F) Flaps were placed on the defect, ensuring the integrity of the sutures and the planned symmetry. (G) Flaps were sutured and placed on the defect, resolving the initial injury. (H) Closing of the oral commissure with a 1 cm extension compared to the right oral commissure from the midline. Illustration on top adapted from: Garritano F, Carr M. Oral commissure bums in children. OperativeTechniques in Otolaryngology-Head and Neck Surgery, 2015; 26, 136-142. Available from: https://www.optecoto.com/article/S1043-1810(15)00072-X/fulltext (Adapted by: Dr. Maria Alejandra Amín Rojas).

A musculocutaneous flap was designed from the adjacent inner cheek, preserving viable orbicularis oris fibers to restore muscular continuity. The upper and lower components of the flap were advanced toward the newly marked commissural apex. The muscular layer was approximated with absorbable sutures to reestablish sphincter function, followed by mucosal approximation to recreate the mucocutaneous junction and define the commissural angle (Fig. 2A–F).

For reconstruction of the lip’s vermilion, the mucosa was everted (“rolled out”) and sutured to reestablish the vermilion border and maintain continuity. Closure was achieved without tension using multifilament absorbable sutures, achieving complete defect coverage and commissural alignment (Fig. 2G and H).

**Figure 3.**
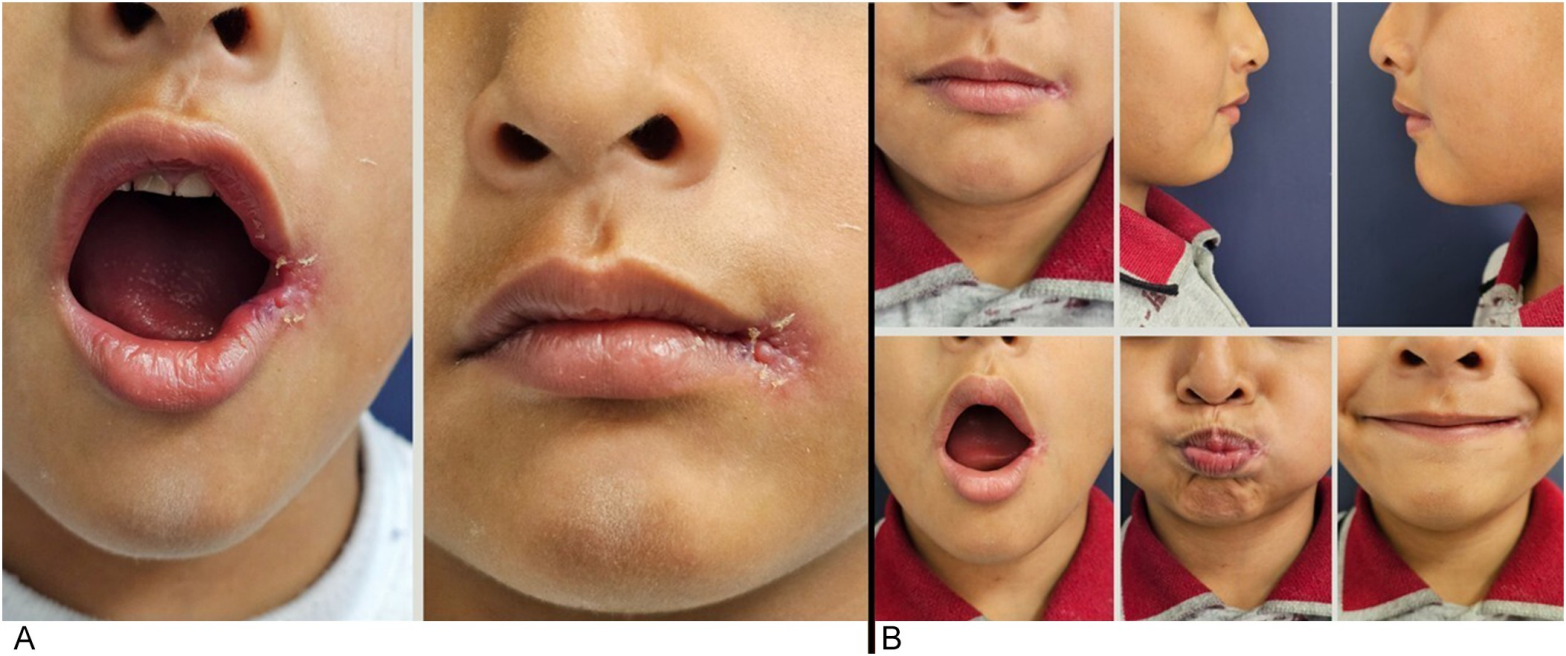
(A) Flap integration after 30 days, showing symmetry improvement and an adequate oral aperture. (B) Symmetry, oral aperture and closure, sphincter function, and smile were preserved after 6 months.

At 1-month follow-up, the patient demonstrated adequate oral aperture, satisfactory symmetry, and hermetic closure of the oral commissure at rest. Preservation of commissural contour and progressive scar remodeling were observed (Fig. 3A). At the 6-month follow-up, edema had decreased, with improved contour of the mouth and labial commissure, and appropriate scar remodeling. Oral function and symmetry were satisfactory with the mouth closed, demonstrating preserved sphincter competence, although a mild residual asymmetry was noted during mouth opening, which was considered acceptable given the initial defect (Fig. 3B).

At the 6-month follow-up, the patient’s mother expressed high overall satisfaction with the outcome. She reported that any residual asymmetry was not noticeable unless specifically pointed out and was limited to a mild asymmetry during mouth opening. She also emphasized the marked visual improvement compared with the initial defect at presentation to our institution.

Written informed consent for the publication of the case details and clinical images was obtained from the patient’s legal guardian.

## Discussion

Electrical burns affecting the perioral region may appear limited in surface area, yet in some cases, they can result in full-thickness defects with relevant functional implications. Unlike extensive burns, where the primary concern is tissue viability, commissural defects pose a distinct reconstructive challenge centered on the restoration of oral competence, symmetry, and muscular continuity^[^6,8^]^. Even minor injuries in this subunit can result in microstomia, drooling, distortion of the vermilion border, and long-term esthetic sequelae. In pediatric patients, these outcomes are amplified by ongoing facial growth and the psychosocial impact of visible deformity^[^6,9^]^.

This case involved a full-thickness labial commissure defect with orbicularis oris muscle exposure, causing incomplete oral closure and disruption of the commissural subunit. Given the unpredictable depth of injury characteristic of electrical burns[8], initial conservative wound management to allow clearer definition of tissue viability was implemented. An early approach with hydrogel-based dressings facilitates autolytic debridement by maintaining a moist microenvironment that promotes autolytic breakdown of necrotic tissue while preserving viable structures^[^10,11^]^. In electrical burns with uncertain zones of stasis, this strategy may improve tissue demarcation, reduce trauma during dressing changes, and decrease the need for repeated debridement prior to definitive reconstruction.

Once tissue viability and defect extent were defined, the reconstructive strategy became the primary consideration. Local tissue rearrangement remains the first-line option, particularly in pediatric patients, due to optimal tissue match, preservation of vascularity, and low donor-site morbidity[8].

The flaps based on the facial artery are a versatile option for small-to-moderate defects, allowing intraoral reconstruction while avoiding visible cutaneous scars when performed early. However, their limited flap width and the potential technical constraints when reconstruction is delayed after injury should be carefully considered during surgical planning^[^12,13^]^. Other alternatives for reconstruction include cross-lip flaps, such as the Abbe and Estlander techniques, which represent established options for full-thickness lip and commissural defects, providing reliable restoration of vermilion continuity and oral competence. Their main limitations include the need for staged procedures with secondary pedicle division and may be associated with temporary functional limitation or residual asymmetry^[^14–16^]^. Similarly, sphincter-preserving approaches such as the Karapandzic flap and large advancement techniques, including Bernard–Burow or Bernard–Webster, can restore extensive defects but carry an increased risk of postoperative microstomia and technical complexity^[^17–19^]^. On the other hand, although tongue flaps may provide acceptable outcomes for selected lip and oral commissure defects, they can be associated with temporary difficulties in speech and oral feeding. More importantly, they usually require a second-stage procedure for flap division approximately three weeks later, exposing the patient to an additional operation and requiring postoperative cooperation during the period before flap division, which may be challenging in pediatric patients^[^20,21^]^. In contrast, the whale-tail–shaped mucocutaneous flap used in the present case allowed a single-stage, anatomically oriented reconstruction using like-with-like tissue, achieving functional restoration while minimizing morbidity and avoiding secondary procedures, highlighting the importance of individualized flap selection based on defect characteristics and patient-specific factors.

A previously reported pediatric case of electrical burn involving the oral commissure was managed with early conservative treatment and prolonged use of a microstomia prevention device. In their report, splint therapy was maintained for approximately 7 months, requiring interventions for appliance placement, adjustment, and eventual scar revision[9]. In contrast, the present case was managed with anatomical reconstruction of the commissural complex in a single operative stage, restoring muscular continuity and mucosal architecture without the need for prolonged splinting or staged procedures. Although preventive splint therapy remains a valid and effective strategy, this case suggests that early reconstruction may achieve functional stability and commissural symmetry in acute full-thickness defects with preserved adjacent viable tissue.

Sequelae of perioral burns are largely related to scar contracture and the progressive reduction of the oral aperture. Microstomia has been described as a frequent and functionally limiting consequence following facial and electrical burns, which is particularly devastating in pediatric populations due to their smaller oral aperture and increased predisposition to hypertrophic scarring[22]. Clinical evidence of microstomia has been documented as early as hospital discharge[23]. Various surgical techniques for post-burn microstomia have been reported, often requiring staged procedures or prolonged adjunctive measures to maintain commissural width^[^22,24,25^]^. At 6 months follow-up, our patient maintained adequate oral aperture, symmetric commissural anatomy, and preserved orbicularis function, without clinical evidence of contracture. However, this follow-up remains relatively short in the context of pediatric facial growth, and longer surveillance is necessary to detect late scar contracture or growth-related asymmetry during adolescence or adulthood in a distant follow-up.

## Conclusion

Oral commissure reconstruction after electrical injury requires precise restoration of muscular continuity, vermilion architecture, and oral competence to preserve long-term function and symmetry. In this case, a whale-tail–shaped mucocutaneous flap enabled single-stage anatomical reconstruction based on like-with-like tissue, achieving stable commissural alignment and functional preservation without needing additional surgeries; nevertheless, because this was a pediatric patient, continued follow-up into adolescence is warranted to monitor facial growth, scar maturation, oral aperture, and the possible recurrence of commissural contracture. Although the reconstructive endeavor prioritizes long-term solutions, this report highlights that the optimal approach is not always the most technically complex. When local tissue allows, a carefully designed local flap can provide a durable, functional, and esthetically satisfactory result in one stage, which is valuable in pediatric patients where limited cooperation and tolerance to prolonged postoperative measures may influence the overall treatment burden.

## Data Availability

Not applicable.
